# Potential Impact of Co-Infections and Co-Morbidities Prevalent in Africa on Influenza Severity and Frequency: A Systematic Review

**DOI:** 10.1371/journal.pone.0128580

**Published:** 2015-06-11

**Authors:** Adam L. Cohen, Meredith McMorrow, Sibongile Walaza, Cheryl Cohen, Stefano Tempia, Marissa Alexander-Scott, Marc-Alain Widdowson

**Affiliations:** 1 Influenza Division, Centers for Disease Control and Prevention, Atlanta, Georgia, United States of America; 2 Influenza Program, Centers for Disease Control and Prevention—South Africa, Pretoria, South Africa; 3 United States Public Health Service, Rockville, Maryland, United States of America; 4 Centre for Respiratory Diseases and Meningitis, National Institute for Communicable Diseases, Sandringham, South Africa; 5 School of Public Health and Faculty of Health Science, University of the Witwatersrand, Johannesburg, South Africa; 6 Division of Applied Research and Technology (DART), National Institute for Occupational Safety and Health (NIOSH), Centers for Disease Control and Prevention, Cincinnati, Ohio, United States of America; 7 University of Illinois, Springfield, Illinois, United States of America; University of Hong Kong School of Public Health, HONG KONG

## Abstract

Infectious diseases and underlying medical conditions common to Africa may affect influenza frequency and severity. We conducted a systematic review of published studies on influenza and the following co-infections or co-morbidities that are prevalent in Africa: dengue, malaria, measles, meningococcus, *Pneumocystis jirovecii* pneumonia (PCP), hemoglobinopathies, and malnutrition. Articles were identified except for influenza and PCP. Very few studies were from Africa. Sickle cell disease, dengue, and measles co-infection were found to increase the severity of influenza disease, though this is based on few studies of dengue and measles and the measles study was of low quality. The frequency of influenza was increased among patients with sickle cell disease. Influenza infection increased the frequency of meningococcal disease. Studies on malaria and malnutrition found mixed results. Age-adjusted morbidity and mortality from influenza may be more common in Africa because infections and diseases common in the region lead to more severe outcomes and increase the influenza burden. However, gaps exist in our knowledge about these interactions.

## Introduction

Until recently, little was known about influenza in Africa except for outbreaks reporting a high case-fatality rate [1–4]. In response to threats from avian and pandemic influenza, surveillance has expanded in recent years, and from 2006–2010, influenza surveillance sites increased more than tenfold across the continent [5]. Recent studies have suggested that influenza is common in Africa. Much of the global burden of severe influenza is in less developed countries [6], and during the 2009 influenza pandemic, Africa may have borne a disproportionate number of deaths from influenza [7].

There are a number of reasons why sub-Saharan Africa specifically may share a disproportionate burden of severe disease outcomes from influenza and should be a focus of interventions to reduce the global burden of disease. One of these is the high prevalence of co-infections and co-morbidities that may be risk factors for more severe influenza illness, such as HIV and tuberculosis [8]. Another important co-infection may be dengue, which is under-recognized and underreported in Africa; it is estimated that as many as 1 million cases occur in Africa annually and 22 countries have reported sporadic cases or outbreaks [9]. Of the more than 600,000 deaths from malaria in 2012, 90% occurred in sub-Saharan Africa [10]. Measles is common in Africa, and *N*. *meningitidis* causes recurring meningitis epidemics in the sub-Saharan meningitis belt. *Pneumocystis jirovecii* pneumonia (PCP) is the most common opportunistic infection among HIV-infected individuals and may be a common cause of respiratory illness in much of sub-Saharan Africa where HIV is prevalent [11]. In 2010, more than 230,000 infants were estimated to be born with sickle cell disease in sub-Saharan Africa, and more than 3.6 million infants were born with sickle cell trait, more than two-thirds of the global burden of disease [12]. The World Health Organization estimates that malnutrition contributes to a third of all deaths in children under five years, and many of these deaths are in sub-Saharan Africa [13].

Little is known about whether co-infections and co-morbidities which are prevalent in Africa have the potential to increase the frequency or severity of influenza infection or to affect response to influenza vaccination. Co-infection of influenza with HIV, tuberculosis, and pneumococcus has been established as causes of severe disease in Africa and have been explored recently in the literature [14–18]. The impact of underlying medical conditions such as diabetes mellitus and asthma on influenza has been explored extensively in the global literature [19]. We conducted a systematic review of published studies to fill the gaps in knowledge of other co-infections and co-morbidities that could also plausibly influence influenza burden in Africa.

## Materials and Methods

We conducted systematic reviews of the literature published from January 1900 to August 2013. Seven databases were searched: Medline [Ovid], Embase, PsycINFO, CINAHL, Web of Science, Cochrane, Global Health, Google Scholar, and CAB Abstracts. Studies were included if they contained original data on the following:

1. Frequency or burden of influenza and co-infection or co-morbidity
2. Disease association between influenza and co-infection or co-morbidity
3. Clinical presentation of influenza and co-infection or co-morbidity
4. Effectiveness of interventions preventing laboratory-confirmed influenza among individuals with the co-infection or co-morbidity

Eligible studies had to be conducted in humans and could be of any study design except case studies. Summary measures gathered as part of the review differed by study design; when available, relative risks, odds ratios (OR), and percents of seroconversion or seroprotection after influenza vaccination were abstracted. Studies could be conducted in any country or setting but needed to be written in English, Spanish, French, Portuguese, Italian, or German.

Databases were last searched in September 2013. The titles of all studies identified through the database searches were reviewed by two reviewers; the abstracts and full text of potentially relevant articles were then reviewed. Additional articles were identified from reviewing bibliographies of published articles.

Seven separate systematic reviews were conducted and reported according to PRISMA Statement guidelines [20]. The topics of the reviews were chosen among infections and morbidities with the following two characteristics: (1) they are prevalent or emerging infections or underlying medical conditions in African countries, and (2) an association with influenza is plausible but not well-accepted or recently reviewed in the literature. The topics of the reviews included in this paper were not intended to be comprehensive nor specific to Africa; they were chosen to fill in research gaps for conditions for which little is known and which might impact influenza in Africa. The seven infections and morbidities in this review were dengue, malaria, measles, meningococcal disease, PCP, hemoglobinopathies, and malnutrition. For each search, we included (1) a term for influenza (“influenza” or “flu”) and (2) a term for each co-infection or co-morbidity, as listed below. Variations on words were also included, e.g., “parasite” and “parasitology,” depending on the database. The search strategy differed slightly depending on the database. Search terms for co-infections and co-morbidities included in this review were as follows:

1. Malaria: malaria, *Plasmodium* or parasite
2. Malnutrition: Malnutrition, kwashiorkor, marasmus, or nutrition
3. Hemoglobinopathies: Hemoglobinopathy, sickle cell, hemoglobin SC, hemoglobin C, or thalassemia
4. Meningococcal Disease: *Neisseria meningitides* or meningococcus
5. Dengue: Dengue
6. Measles: Measles
7. *Pneumocystis jirovecii* pneumonia: *Pneumocystis*

Several studies were identified that assessed probiotics or other nutritional supplements not traditionally found in resource-limited settings; therefore, we restricted inclusion criteria for these malnutrition articles to nutritional supplements that are commonly available in African settings such as zinc sulfate or Vitamin A. When appropriate, we rated the quality of the individual studies using the Newcastle-Ottawa Scale [21]. Studies were considered high quality if the Newcastle-Ottawa Scale was ≥7 out of 9; studies were of low quality if the score was ≤3 of 9. Individual studies were also assessed for potential bias or confounding. No review protocol was registered. Human subjects review was not necessary since this was a systematic review of published studies.

## Results

### Co-infections

#### Dengue

**Fig 1:**
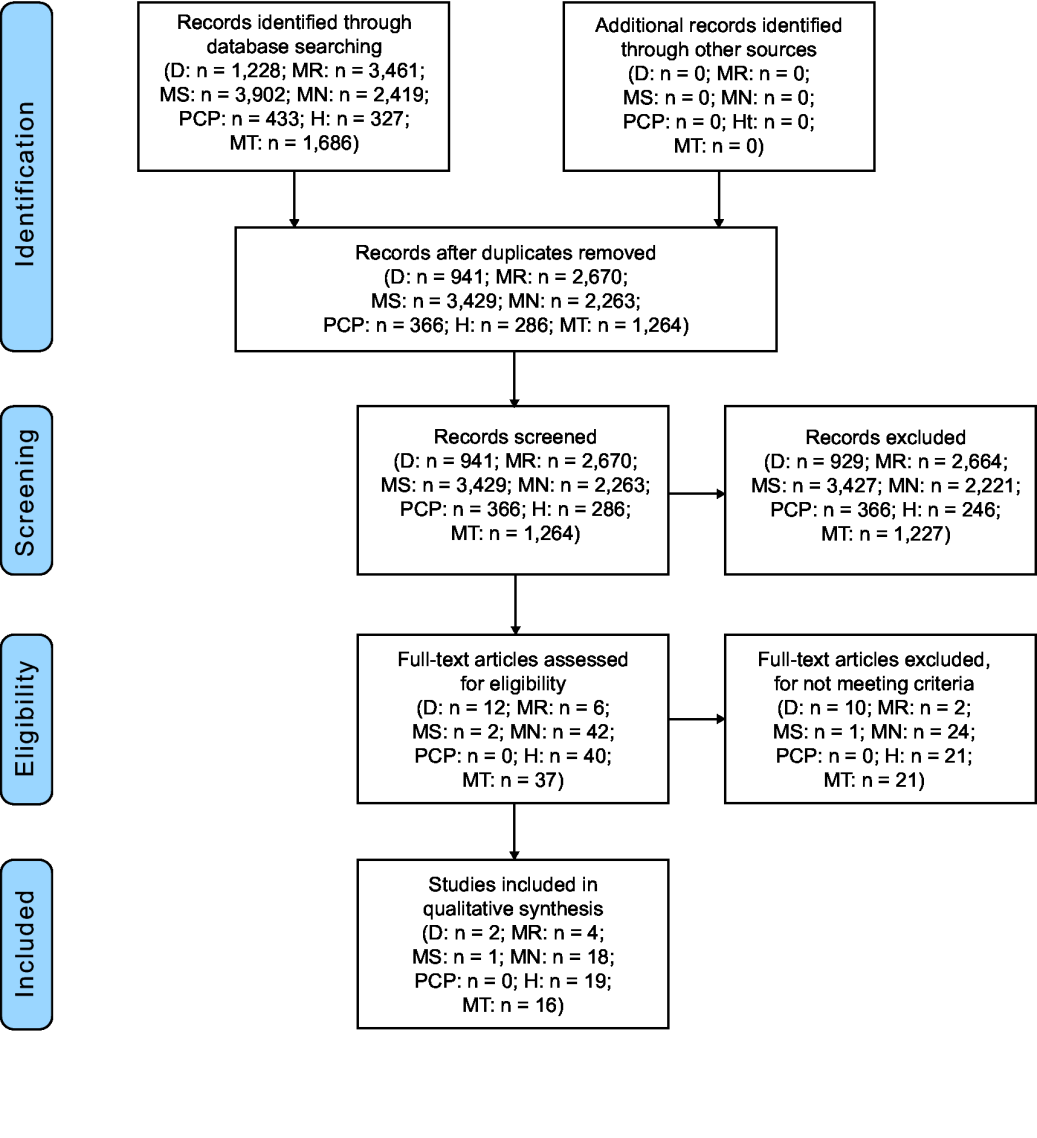
PRISMA flow diagram for systematic reviews of influenza and dengue (D), malaria (MR), measles (MS), meningococcus (MN), *Pneumocystis jirovecii* pneumonia (PCP), hemoglobinopathies (H), and malnutrition (MT).

Our literature search yielded 1,228 articles of which 287 were duplicates (Fig 1). Of the remaining 941 articles, 12 were deemed to be potentially relevant and were fully assessed for eligibility and 2 met selection criteria, both from Nicaragua. Both studies were large cohort studies of high quality (S1 Table). The 2009 pandemic influenza A(H1N1)pdm09 (pH1N1) coincided with the dengue season in Nicaragua, and both studies found that co-infection was associated with increased illness severity. The first study followed a cohort of children for influenza A(pH1N1) infection. Of 185 children with influenza A(pH1N1) infection, 3 were co-infected with dengue. All three (100%) of co-infected children were hospitalized, compared with 1% of those with influenza A(pH1N1) infection only (p<0.001) and 42% of those with dengue infection only (p = 0.08) [22]. The second study followed a cohort of children with dengue and found that 66% had antibodies to influenza A(pH1N1), suggesting recent infection with influenza. Dengue patients with serologic evidence of recent past influenza A(pH1N1) infection had nearly twofold increased odds of developing shock compared with dengue patients without evidence of recent influenza A(pH1N1) infection [OR = 1.93, 95% confidence interval (CI) 1.01–3.31, p = 0.045] [23].

#### Malaria

Titles and abstracts of 3,461 published articles were reviewed (Fig 1). Of these articles, 669 were duplicates and were removed. The remaining 2,670 titles and abstracts were assessed and 4 articles met the inclusion criteria for this review. All 4 studies assessing malaria and influenza co-infection were from Africa: 2 from Kenya, and 1 each from Senegal, and Tanzania. Malaria infection was diagnosed by blood smear in all studies. Three of the four studies were of high quality, and all included at least 25 cases (S1 Table).

The first three studies evaluated the risk of co-infection, but not the impact on disease severity. The earliest of these studies, which was from Tanzania and had the lowest quality, used serology to confirm influenza infection in 14 of 24 subjects, which was a subset of 128 subjects clinically diagnosed with influenza [24]. Among children aged 0–9 years, 28 (62%) of 45 with influenza also had malaria parasitemia. Among age- and village-matched asymptomatic control children, 38 (84%) of 45 had parasitemia, which was a significantly higher parasitemia (p = 0.02) and a mean positive parasite density than influenza cases (p = 0.01). No statistically significant differences were identified in older age groups. The remaining studies all used reverse transcription polymerase chain reaction to identify influenza infections. A study of children aged 5–10 years in Kenya found that 4 (11%) of 36 subjects with influenza infection were co-infected with malaria [25]. Likewise, influenza infection occurred in 4 (13%) of 31 children with malaria vs 32 (40%) of 81 children without malaria infection (p = 0.04). A study in Senegal found that among 82 episodes of acute respiratory infection in 67 children, 1 (4%) of 25 with influenza had a malaria co-infection, while 6 (18%) of 34 children with no respiratory virus identified had malaria (p = 0.11) [26]. Another study in Kenya found 45% (149/331) of influenza-positive patients were co-infected with malaria, whereas only 6% (149/2408) of malaria-positive patients were co-infected with influenza [27]. Children hospitalized for respiratory disease and with influenza infection were less likely than those without influenza to have malaria (risk ratio [RR], 0.57–0.76 across sites and ages), and children with malaria infection were less likely than those without to have influenza (RR, 0.36–0.63); these findings may be because febrile children are likely to have influenza or malaria infection, but not necessarily co-infection with both. The authors also evaluated the severity of co-infection in this study. Among co-infected children aged 24–59 months, hospital length of stay was 2.7 and 2.8 days longer than influenza-only-infected children at the 2 sites, and 1.3 and 3.1 days longer than those with malaria only (all p < 0.01).

#### Measles

Our literature search yielded 3,902 articles (Fig 1). Of these, 483 were duplicates, leaving 3,429 articles. The full text of two was reviewed, and one met selection criteria, which was of low quality (S1 Table). This study from Italy in the 1960’s tested 68 patients with measles for influenza co-infection, as well as other respiratory pathogens. Complications such as pneumonia or enteritis were found in 60% (9/15) of patients with measles and influenza A, 63% (5/8) of patients with measles and influenza B, but only 17% (5/30) of patients with measles and no identified respiratory viral co-infection (p = 0.03 for patients with measles and any influenza compared with measles and no viral co-infection) [28].

#### Meningococcus

Our literature search yielded 2,419 articles, of which 156 were duplicates (Fig 1). This yielded 2,263 articles; the full text of 42 were screened for eligibility, and 18 met selection criteria. Of these, half (9) were of high quality and three were of low quality (S1 Table). Six used an ecological cohort study design, though none of those were of low quality.

The association between invasive meningococcal disease and antecedent viral infection was noted as long ago as World War I [29], though the patients in these early reports were not uniformly tested for influenza and meningococcus and a definitive association was not found. More recent studies from a variety of settings have observed a temporal association between influenza and meningococcal meningitis outbreaks: influenza B in India [30], influenza A and B in Germany [31], and influenza A in the United Kingdom [32]. Temporal associations have been confirmed by testing patients with meningococcal disease for serologic evidence of recent influenza infection. During concurrent outbreaks of influenza and meningococcal disease at a mental hospital in the U.S. in 1968, patients with meningococcal disease serogroup B were more likely to have a fourfold or greater rise in antibody titers to influenza A than controls [33]. Similarly, patients with meningococcal disease in England and Wales in 1989 were nearly four times more likely than age-matched controls to have serologic evidence of recent influenza A infection as measured by high antibody titers (OR 3.9, 95% CI 1.2–13.9) [34]. Two more recent outbreaks also confirmed an association in settings where close contact likely perpetuated transmission: a cluster of 5 matching, nongroupable meningitis cases in children who all rode the same school bus in the U.S. had increased risk of serologic evidence of influenza B infection as measured by high titers [35], and an outbreak of 9 cases of meningococcal serogroup C infections among Greek Air Force recruits had evidence of influenza B infection, either by a four-fold increase in antibody titers of acute and convalescent serum samples or a permanent high antibody titer of ≥1:64 in both serum samples [36].

Some studies have looked for evidence of any viral respiratory infection among patients with meningococcal disease outside the setting of acute outbreaks. One study found an association between a four-fold rise in influenza B titers and bacterial meningitis among children admitted to a hospital in the U.S.; however, this association was not specific for bacterial meningitis caused by *N*. *meningitidis* [37]. A similar association has been identified among students at an Air Force base in the U.S. who had meningococcal serogroup C or Y disease [38].

More recently, a number of studies have quantified the magnitude of the ecologic associations between the timing of influenza and meningococcal epidemics in a number of settings through modelling of surveillance data from many years (Table 1). Although there has been one study that found no ecologic association between influenza and meningococcal disease [39], the majority of these studies found a statistically significant association [40–45]. One study found an association only among infants less than 1 year of age [40] and another only with meningococcal disease serogroup B [44]. Studies from Canada and Germany have found an association between influenza A and invasive meningococcal disease that was strongest with a 1-week lag between influenza and meningococcal disease [42, 43].

**Table 1:** Summary of ecologic modelling studies evaluating the association between influenza and invasive meningococcal disease (IMD).

Reference	Location	Time period	Influenza included	Meningococcal disease included	Modeling technique	Findings	Additional information
Moreno-Civantos, *et al*. (2000) [45]	Spain	1964–1997	Influenza A and B	IMD	Time-series regression using Box-Jenkins methodology	Positive correlations between influenza and meningococcus were found at lags of 0, 1, 2, and 3 weeks	None
Jensen, *et al*. (2004) [40]	Denmark	1980–1999	Influenza A and B	IMD	Log-linear Poisson regression	29.4 (6.1–57.8) percent increase in number of IMD cases per 100 influenza cases per 100,000 population in children <1 year of age	Association for other age groups was in same direction as found for those <1 year of age but not statistically significant
Domínguez, *et al*. (2007) [44]	Catalonia, Spain	1996–2004	Influenza Influenza A and B	IMD serogroup B	Log-linear Poisson regression	1.64 (1.31–2.06) relative risk	Association not seen with invasive meningococcal disease serogroup C
Paul, *et al*. (2008) [42]	Germany	2001–2006	Influenza A and B	IMD	Negative binomial regression	Significant association between influenza and IMD (rate and odds ratio not given)	Highest association with 1 week lag between influenza and IMD, closely followed by no lag.
Jansen, *et al*. (2008) [41]	Netherlands	1997–2003	Influenza A and B	IMD	Spearman correlation coefficient, using influenza active and non-active periods	1.51–1.80 incidence rate ratio of periods of influenza virus predominance vs. summer baseline	Association varies slightly based on age
Tuite, *et al*. (2010) [43]	Ontario, Canada	2000–2006	Influenza A	IMD	Negative binomial regression	1.18 (1.06–1.31) incidence rate ratio of IMD per 100 case increase of influenza	Associated with 1 week lag between influenza A and IMD; no association with influenza B
Tuite, *et al*. (2010) [43]	Ontario, Canada	2000–2006	Influenza A	IMD	Case-crossover	2.03 (1.28–3.23) increased odds ratio of IMD per 100 case increase of influenza	None
Allard, *et al*. (2011) [39]	Montreal, Canada	1996–2008	Influenza A and B	IMD	Negative binomial regression	No significant association with up to a 5 week lag between influenza and IMD	None

IMD = Invasive meningococcal disease.

In summary, there is evidence from a number of observational studies, many of high quality, that meningococcal disease increases in frequency following influenza.

#### *Pneumocystis jirovecii* pneumonia (PCP)

Our literature search yielded 433 articles; 66 duplicates were removed, leaving 366 articles, of which none met selection criteria (Fig 1).

### Co-morbidity host factors

#### Hemoglobinopathies

The literature review identified 327 potentially relevant articles, of which 41 were removed as duplicates (Fig 1). We reviewed the titles and abstracts of the remaining 286 manuscripts, and assessed the full text of 40 articles for eligbility, of which 19 met selection criteria. None were from the African region, and all studied sickle cell disease except one. Six studies were immunogenicity studies so the Newcastle-Ottawa Score was not applicable. Of the remaining studies, 7 were of high quality, 1 was of good quality, and 5 were of low quality.

Four published case series of influenza infection among patients with sickle cell disease showed that influenza can precipitate acute chest syndrome among individuals with sickle cell disease [46–49] and that influenza A(pH1N1) may have caused more severe outcomes than seasonal influenza when it was first identified [46]. A case series of 75 children hospitalized with influenza A(pH1N1) in Milwaukee, Wisconsin, USA, found 16% had a hemoglobinopathy [50]. In 2009, an outbreak of influenza A(pH1N1) occurred at a camp in the U.S. for children with hematologic and oncologic conditions; although there was no comparison group, children with sickle cell disease were disproportionately (82%) affected by influenza, and most (89%) were hospitalized [51]. A study from Brazil found that sickle cell disease was not associated with death among patients infected with influenza A(pH1N1) [52]. A study from Spain found that 3% of children seen in an emergency department with influenza A(pH1N1) had a hemoglobinopathy or underlying blood disorder, and that those conditions were associated with a nearly six-fold increased odds of admission [53]. The largest and most rigorous study of the burden of influenza among individuals with sickle cell disease used administrative data from four U.S. states and found that children with sickle cell disease were hospitalized with influenza at 56 times the rate of children without sickle cell disease [54].

Patients with hemoglobinopathies have impaired immune responses, and individuals with sickle cell anemia may develop respiratory complications such as acute chest syndrome in response to influenza infection. Six studies evaluated whether influenza vaccine is immunogenic among individuals with sickle cell disease (Table 2). Five studies found that influenza vaccine was immunogenic among children and adults with seroconversion rates of 60–87% and seroprotection rates of 70–100% [55–59]; however, the immune response may be blunted in children who receive chronic transfusions (90% immune response among subjects not on chronic transfusions compared with 61% among those subjects receiving chronic transfusions) [59]. One study in adults with sickle cell disease found impaired immune responses to trivalent inactivated vaccine, suggesting that the long-term effects of sickle cell disease, such as splenic atrophy, may affect immune response to vaccination [60]. Influenza vaccines are generally considered safe among individuals with sickle cell disease; however, one study suggested a 7- to 9-fold increased risk of hospitalization for sickle cell anemia after administration of trivalent inactivated vaccine [61]. Follow-up studies by the same group did not find increased hospitalizations for sickle cell crises in adults [62] or children [63] after influenza vaccination. No vaccine effectiveness or efficacy studies among persons with sickle cell disease have been conducted.

**Table 2:** Summary of studies evaluating influenza vaccine among individuals with hemoglobinopathies. Seroprotection was defined as >1:40 HI titers.

Reference	Location	Population	Vaccine	Findings	Additional information
Steinberg, *et al*. (1978) [55]	California, United States	Children 4–18 years of age with sickle cell disease	Bivalent influenza A split virus (subvirion)	90% seroprotection following 2 doses	None
Glezen, *et al*. (1983) [56]	Texas, United States	Children <5 years of age with sickle cell disease	Trivalent inactivated	68–84% of school age children had titers ≥1:32 following 1 dose; 55–73% of preschool age children had titers ≥1:32 following 2 doses	None
Ballester, *et al*. (1985) [60]	Michigan, United States	Adults with sickle cell disease	Trivalent inactivated vaccine	50% had decreased or undetectable influenza IgM following vaccination	Patients also had decreased splenic function
Souza, *et al*. (2010) [57]	Brazil	Children 8 years and older and adults with sickle cell disease	Trivalent inactivated virosome-adjuvanted	60–84% seroconversion and 70–100% were seroprotection to all 3 strains	No statistically significant differences between the two vaccines
Souza, *et al*. (2010) [57]	Brazil	Children 8 years and older and adults with sickle cell disease	Trivalent inactivated split	62–68% seroconversion and 77–98% were seroprotection to all 3 strains	
Esposito, *et al*. (2010) [64]	Italy	Adolescents and adults with β-thalassemia major	Monovalent influenza A(pH1N1)MF59-adjuvanted	87% seroconversion and 100% seroprotection 4 weeks after dose	None
Long, *et al*. (2012)	New York, United States	Children with sickle cell disease	Monovalent influenza A(pH1N1)	70% seroprotection approximately 6 weeks following vaccination; 79% cell-mediated immunity responses as measured by IFNγ ELLISPOT assay	Hydroxyurea use was associated with lessened HI antibody response
Purohit, *et al*. (2012) [59]	Florida, United States	Children with sickle cell disease	Monovalent influenza A(pH1N1)	76% seroprotection 8 month after vaccination	Hydroxyurea use was not associated with lessened HI antibody response; chronic transfusions was

HI = hemagglutinin inhibition.

We found only one article that evaluated the interaction between influenza vaccine and thalassemia. This study found that the 2009 influenza A(pH1N1) MF59-adjuvanted vaccine was immunogenic and safe among adolescents and young adults with β-thalassemia [64].

In summary, a number of high quality observational studies showed that sickle cell disease is associated with increased severity and frequency of influenza. Immunogenicity studies suggest that influenza vaccine is effective among those with sickle cell disease.

#### Malnutrition

Titles and abstracts of 1,686 published articles were reviewed (Fig 1). Of these articles, 422 were duplicates and were removed. The remaining 1,264 titles and abstracts were assessed using the above criteria. 37 original articles were assessed in full for eligibility and 16 review articles were reviewed for additional references. Among the 16 articles that met the inclusion criteria for this review, 5 assessed the impact of malnutrition in children on influenza disease and 11 assessed malnutrition as a risk factor for poor response to influenza vaccination in the elderly. Two studies were randomized trials, so the Newcastle-Ottawa Score was not applicable. Of the remaining studies, eight studies were of high quality and none were low quality (S1 Table).

A number of studies have shown that protein-energy or micronutrient malnutrition may modify the clinical course of influenza infection in children. Two studies assessed malnutrition during the influenza A(pH1N1) pandemic period. A study of hospitalized children in Argentina found that malnutrition was a risk factor for death among children with influenza A(pH1N1) infection [RR = 3.07 (1.46–6.48)] [65]. A second study of 74 children hospitalized with influenza A(pH1N1) in Peru found that malnutrition was common (40%) but was not associated with severe disease [OR = 1.7 (0.4–7.2)]; in this study, the type of malnutrition was not specified [66]. A third study among Gambian children that found few cases of influenza among malnourished children presenting to care at an outpatient clinic (≤5% prevalence) and no statistically significant difference in the prevalence of influenza between malnourished and nourished children [67]. A randomized trial of vitamin D supplementation in Japanese children receiving 1200 IU Vitamin D3 daily found a reduced risk of influenza A infection (RR = 0.58, 95% CI 0.34–0.99) [68]. Lastly, a U.S. study that examined the impact of body mass index (BMI) on influenza A(pH1N1) found that among children 2–19 years of age hospitalization was associated with being underweight (BMI ≤5^th^ percentile) among those with [OR = 12.5, (3.4–45.5)] or without [OR = 5.5, (1.3–22.5)] chronic conditions [69].

Among the elderly there were 11 studies that assessed malnutrition and nutritional supplementation as a risk factor for poor immunogenic response to vaccination, as measured by humoral immunity. Four articles assessed the relationship between general nutritional status and BMI and response to influenza vaccine [70–73], but only 1 study found an association between poor vaccine response and low BMI [73]. Other studies found statistically significant associations between markers of nutritional status including haemoglobin [71], iron [71], total protein [71], serum albumin [74], vitamin D [75], and vitamin E [76] and poor response to influenza vaccination. Other studies found no association between protein [77], beta-carotene [78], retinol [78], alpha-tocopherol [78] or zinc [78] and response to influenza vaccination. Likewise, two studies of zinc supplementation in the elderly found conflicting results [79, 80]. One study found that neither zinc nor zinc plus arginine affected response to influenza immunization in the elderly [79], while the other found that supplementation with zinc sulphate and selenium sulfide increased seroprotection rates to influenza A(H3N2) [80].

In summary, there were a number of good or high quality studies, but the results were mixed regarding whether there was an association between influenza and malnutrition.

## Discussion

This systematic review suggests that infections and diseases more prevalent in settings such as Africa may affect the frequency and severity of influenza infection (Table 3). Studies included in this review suggest an association between influenza severity or frequency and sickle cell disease, dengue, and measles, though the data on the potential interaction between influenza and dengue and measles are based few studes and the study of measles is of low quality. Influenza infection increased the frequency of meningococcal disease. The studies on malaria and malnutrition found mixed results with respect to association with influenza frequency and severity. Other infections not included in these systematic reviews, such as HIV, tuberculosis, and pneumococcal disease, are associated with increased severity of influenza. We found no data on influenza and PCP. Many of the studies were not of high quality. More high quality studies should be conducted in lower income settings and African countries to confirm these associations. Although influenza has long been under-recognized as a cause of illness and death in Africa, this review suggests that prevention and vaccination against influenza can prevent severe influenza infection in these high-risk and co-infected groups.

**Table 3:** Associations of co-infections and co-morbidities prevalent in Africa with increased severity or incidence of influenza.

Co-infection or co-morbidity	Evidence of association with increased severity of influenza	Evidence of association with increased frequency of influenza	Evidence that influenza increases the frequency of the co-infection or co-morbidity	Systematic review included in this analysis
Dengue	Yes [22, 23]	No studies	No	Yes
Hemoglobinopathies, namely sickle cell disease	Yes (associated with hospitalization[53] but not death[52])	Yes [50, 51, 54]	No	Yes
HIV	Yes [8, 14, 82, 87]	Yes [8, 14, 82, 83]	No	No
Malaria	Yes [27]	No [24–27]	No	Yes
Malnutrition	Unclear (one study found an association [65] and one did not [66])	Unclear (two studies found an association [68, 69] and one did not [67])	No	Yes
Measles	Yes [28]	No studies	No	Yes
Meningococcal disease	No studies	No	Yes [30–38, 40–45] (One study[39] did not find an association)	Yes
Pneumococcal disease	Yes [15, 16]	No	Yes [15, 16]	No
*Pneumocystis jirovecii* pneumonia (PCP)	No studies	No studies	No	Yes
Tuberculosis	Yes [17, 91]	Yes [8, 17, 91]	No	No
Underlying medical conditions, such as diabetes mellitus and asthma	Yes [19]	Yes [19]	No	No

Applicable references from the systematic review and the literature are included.

Although HIV was not included in this review, it is epidemic in many sub-Saharan countries [81], and recent reviews found that HIV and AIDS are associated with increased severity of influenza [14, 82]. In adults in the U.S., HIV is associated with increased severity [83] and mortality [84] among patients with influenza. HIV-infected adults in Kenya have been found to have increased odds of hospitalization for pneumonia [85]. HIV-infected children in South Africa have an increased risk of hospitalization for pneumonia [86, 87]. In South Africa, HIV confers increased odds of death from influenza-associated pneumonia in all ages [8, 88], and the HIV epidemic drives the epidemiology and clinical presentation of influenza [8, 89].

Influenza can be a risk factor for other infections. As has been shown with pneumococcal disease [15, 16], influenza appears to increase an individual’s susceptibility to meningococcal disease. Similarly, tuberculosis is epidemic in many sub-Saharan countries [81], and influenza may be associated with an increased risk of death from tuberculosis [90, 91]. Mortality data from the 1918 influenza pandemic and more recent data from South Africa suggest that death from tuberculosis may be precipitated by co-infection with influenza [17, 91].

Social and environmental factors may increase the risk of severe influenza in settings like Africa. As classified by World Bank, all of sub-Saharan African countries are low- or middle-income countries [92], and lower socioeconomic status is associated with increased severity and burden of influenza disease [93–95]. Chronic diseases such as diabetes mellitus and lung disease are also common in Africa, and may affect influenza [19]. Low birth weight and non-exclusive breast feeding are common in sub-Saharan Africa [81]. Solid fuel use is common in sub-Saharan Africa and leads to exposure to indoor air pollution. Low birth weight, non-exclusive breast feeding and exposure to indoor air pollution are associated with pneumonia, if not with influenza specifically [96–98].

Some infectious diseases common in Africa may make the diagnosis of influenza challenging because of similar clinical presentations. Malaria and dengue can present clinically with febrile syndromes similar to influenza [99], and co-infection has been reported [100–104]. Although measles and PCP are common in Africa, can present as febrile respiratory illness, and may be associated with changes in the immune system, we found too few published articles to comment on the interaction of those infections with influenza. If there is an association between PCP and influenza infection, it may be difficult to tease out the effects of underlying HIV infection since the majority of patients with PCP will also have HIV infection.

The main limitation of these reviews are the paucity of high quality published articles in some areas—namely dengue, measles, and PCP—which limit the conclusions that can be drawn. Nearly all of the studies use an observational study design, including ecological cohort studies of influenza and meningococcus. Although many of these observational studies were of high quality, and randomized controlled trials would not be ethical or feasible to answer many of these research questions, observational studies are subject to biases. Except for the publications on malaria, most of the published articles are from countries not in Africa, so the findings may not be generalizable to low-income and African settings. For example, only one study evaluating the association between meningococcal disease and respiratory viruses has been conducted in Africa; although there was a statistically significant association found, the viruses evaluated (adenovirus, parainfluenza, respiratory syncytial virus, and rhinovirus) did not include influenza [105]. This is also not a comprehensive review of every condition that might impact influenza in Africa. Some of the articles may use less effective laboratory techniques to identify influenza, particularly older studies that were conducted before more sensitive and specific techniques, such as polymerase chain reaction for influenza, were developed or commonly used; this may misclassify some patients with influenza as not having influenza. Studies used differing definitions of immune response, either high titers or a four-fold response, which may make it difficult to interpret and determine active co-infection. Lastly, many of the studies did not control for potential confounding factors such as other infections (e.g., HIV), severity at presentation, and health care access and did not include comparison groups to adequately address the research question.

Vaccination against influenza remains the most important and effective way to prevent disease from influenza. Very little influenza vaccine is available and used in sub-Saharan Africa [106]. Vaccinating populations at high risk of severe complications, such as individuals with HIV infection, tuberculosis, and sickle cell disease, should be prioritized in countries where those conditions are prevalent. Although there are few studies demonstrating the efficacy or effectiveness of influenza vaccine among individuals who are infected with tuberculosis, influenza vaccine is safe and effective among adults that are infected with HIV [107] and those that have sickle cell disease [55–59]. Since influenza can predispose individuals to pneumococcal and meningococcal disease, vaccination against those pathogens may reduce the burden of these secondary bacterial infections and be important in minimizing disease seasonally [108] and during a pandemic [109]. Prevention and treatment of other diseases such as HIV with antiretrovirals will also be an essential component to controlling influenza in settings with high HIV prevalence. Micronutrient supplementation may also be useful, particularly in settings with malnutrition, although micronutrient interventions would be unlikely to be implemented for influenza only. Clinicians must not forget that influenza can cause severe disease in settings such as Africa, and policymakers should consider how to best control the disease in their countries through direct prevention of influenza and other underlying conditions and infections.

## Supporting Information

S1 PRISMA Checklist (DOC)

S1 Table List of studies included in systematic review and their study design, size, and quality. (DOCX)
