# Supplementary material for: Potential Impact of Co-Infections and Co-Morbidities Prevalent in Africa on Influenza Severity and Frequency: A Systematic Review
Source: PLoS One. 2015 Jun 11;10(6):e0128580. doi: 10.1371/journal.pone.0128580 (PMC4466242; doi:10.1371/journal.pone.0128580)
Supplement: S1 Table — (DOCX) [file pone.0128580.s002.docx]

**S1 Table. List of studies included in systematic review and their study design, size, and quality**

| **Study** | **Study design** | **Number of cases, controls, or cohort** | **Newcastle-Ottawa Score (out of a possible score of 9)** |
| --- | --- | --- | --- |
| **Dengue** |  |  |  |
| Gordon, *et al.* (2010) | Cohort | 4391 | 8 |
| Gutierrez, *et al.* (2011) | Cohort | 3711 | 7 |
| **Malaria** |  |  |  |
| Rooth, et al. (1992) | Case-control | 128 cases, 45 controls | 5 |
| Waitumbi, *et al.* (2010) | Case-control | 31 cases, 81 controls | 7 |
| Niang, *et al.* (2010) | Case-control | 25 cases, 57 controls | 7 |
| Thompson, *et al.* (2012) | Case-control | 331 cases, 3721 controls | 7 |
| **Measles** |  |  |  |
| Colonnello, *et al.* (1967) | Case-control | 30 cases, 38 controls | 6 |
| **Meningococcus** |  |  |  |
| Rolleston, et al. (1919) | Cohort | Not given | 0 |
| Lal, *et al.* (1963) | Cohort | Not given | 2 |
| Schubiger, *et al.* (1986) | Cohort | 57 cases | 7 |
| Reilly, *et al.* (1991) | Case-control | 25 cases | 1 |
| Young, *et al.* (1972) | Case-control | 19 cases, 77 controls | 7 |
| Cartwright, *et al.* (1991) | Case-control | 43 cases, 67 controls | 7 |
| Harrison, *et al.* (1991) | Case-control | 5 cases, 59 controls | 7 |
| Makras, *et al.* (2001) | Case-control | 9 cases, 55 controls | 6 |
| Moore, *et al.* (1990) | Case-control | 62 cases, 62 controls | 9 |
| Krasinski, *et al.* (1987) | Case-control | 160 cases, 138 controls | 3 |
| Eickhoff, *et al.* (1971) | Cohort | Not given | 3 |
| Allard, *et al.* (2012) | Cohort (ecologic) | 156 meningococcal cases | 7 |
| Jensen, *et al*. (2004) | Cohort (ecologic) | 413 meningococcal cases | 7 |
| Jansen, *et al.* (2008) | Cohort (ecologic) | 3072 meningococcal cases | 6 |
| Paul, *et al.* (2008) | Cohort (ecologic) | Not given | 6 |
| Tuite, *et al.* (2010) | Cohort (ecologic) | 240 meningococcal cases | 7 |
| Dominguez, *et al.* (2007) | Cohort (ecologic) | Not given | 7 |
| Moreno-Civantos, *et al.* (2000) | Cohort (ecologic) | ~2300 meningococcal cases/year | 6 |
| **Hemoglobinopathies** |  |  |  |
| Strouse, *et al.* (2010) | Case series | 123 | 7 |
| Inusa, *et al.* (2010) | Case series | 2200 | 5 |
| Jacobs, *et al.* (2011) | Case series | 5 | 1 |
| George, *et al.* (2011) | Case series | 48 | 3 |
| Kumar, *et al.* (2010) | Case series | 75 | 3 |
| Morrison, *et al.* (2011) | Cohort | 165 | 7 |
| Ribeiro, *et al.* (2010) | Cohort | 5242 | 1 |
| Lera, *et al.* (2011) | Cohort | 412 | 9 |
| Bundy, *et al.* (2010) | Cohort | 7896 | 7 |
| Steinberg, *et al.* (1978) | Immunogenicity study | 27 | Not applicable |
| Glezen, *et al.* (1983) | Immunogenicity study | 42 | Not applicable |
| Souza, *et al.* (2010) | Immunogenicity study | 90 | Not applicable |
| Long, *et al.* (2012) | Immunogenicity study | 112 | Not applicable |
| Purohit, *et al.* (2012) | Immunogenicity study | 38 | Not applicable |
| Ballester, *et al.* (1985) | Case-control | 23 | 3 |
| Hambidge, *et al.* (2006) | Cohort | 45356 | 8 |
| Hambidge, *et al.* (2011) | Cohort | 2524 | 8 |
| Hambidge, *et al.* (2012) | Cohort | 1085 | 8 |
| Esposito, *et al.* (2010) | Immunogenicity study | 58 | Not applicable |
| **Malnutrition** |  |  |  |
| Gentile, *et al*. (2011) | Cohort | 2367 | 6 |
| Miranda-Choque, *et al.* (2011) | Cohort | 74 | 5 |
| Adegbola, *et al.* (1994) | Case-control | 449 | 7 |
| Urashima, *et al.* (2010) | Randomized controlled trial | 167 cases, 167 controls | Not applicable |
| Morgan, *et al.* (2010) | Case-cohort | 361 hospitalizations, 233 deaths | 7 |
| Bellei, *et al.* (2006) | Cohort | 120 | 5 |
| Fulop, *et al.* (1999) | Cohort | 23 | 6 |
| Potter, *et al.* (1999) | Cohort | 260 | 8 |
| Sagawa, *et al.* (2011) | Cohort | 203 | 9 |
| Hui, *et al.* (2006) | Cohort | 127 | 9 |
| Chadha, *et al.* (2011) | Cohort | 35 | 7 |
| Hara, *et al.* (2005) | Case-control | 153 cases, 95 controls | 7 |
| Pozzetto, *et al.* (1993) | Case-control | 82 cases, 50 controls | 6 |
| Gardner, *et al.* (2000) | Case-control | 61 cases, 27 controls | 6 |
| Provinciali, *et al.* (1998) | Cohort | 384 | 8 |
| Girodon, *et al.* (1999) | Randomized controlled trial | 725 in 4 arms | Not applicable |
